# Selective Continuous Positive Airway Pressure Withdrawal With Supplemental Oxygen During Slow-Wave Sleep as a Method of Dissociating Sleep Fragmentation and Intermittent Hypoxemia-Related Sleep Disruption in Obstructive Sleep Apnea

**DOI:** 10.3389/fphys.2021.750516

**Published:** 2021-11-22

**Authors:** Anna E. Mullins, Ankit Parekh, Korey Kam, Bresne Castillo, Zachary J. Roberts, Ahmad Fakhoury, Daphne I. Valencia, Reagan Schoenholz, Thomas M. Tolbert, Jason Z. Bronstein, Anne M. Mooney, Omar E. Burschtin, David M. Rapoport, Indu Ayappa, Andrew W. Varga

**Affiliations:** Division of Pulmonary, Critical Care, and Sleep Medicine, Mount Sinai Integrative Sleep Center, Icahn School of Medicine at Mount Sinai, New York, NY, United States

**Keywords:** obstructive sleep apnea (OSA), sleep disordered breathing, hypoxic burden, sleep disruption, arousal, desaturation, oxygenation

## Abstract

Obstructive sleep apnea (OSA) is considered to impair memory processing and increase the expression of amyloid-β (Aβ) and risk for Alzheimer’s disease (AD). Given the evidence that slow-wave sleep (SWS) is important in both memory and Aβ metabolism, a better understanding of the mechanisms by which OSA impacts memory and risk for AD can stem from evaluating the role of disruption of SWS specifically and, when such disruption occurs through OSA, from evaluating the individual contributions of sleep fragmentation (SF) and intermittent hypoxemia (IH). In this study, we used continuous positive airway pressure (CPAP) withdrawal to recapitulate SWS-specific OSA during polysomnography (PSG), creating conditions of both SF and IH in SWS only. During separate PSGs, we created the conditions of SWS fragmentation but used oxygen to attenuate IH. We studied 24 patients (average age of 55 years, 29% female) with moderate-to-severe OSA [Apnea-Hypopnea Index (AHI); AHI4% > 20/h], who were treated and adherent to CPAP. Participants spent three separate nights in the laboratory under three conditions as follows: (1) consolidated sleep with CPAP held at therapeutic pressure (*CPAP*); (2) CPAP withdrawn exclusively in SWS (*OSA*_*SWS*_) breathing room air; and (3) CPAP withdrawn exclusively in SWS with the addition of oxygen during pressure withdrawal (*OSA_*SWS*_* + *O*_2_). Multiple measures of SF (e.g., arousal index) and IH (e.g., hypoxic burden), during SWS, were compared according to condition. Arousal index in SWS during CPAP withdrawal was significantly greater compared to CPAP but not significantly different with and without oxygen (*CPAP* = 1.1/h, *OSA_*SWS*_* + *O2* = 10.7/h, *OSA*_*SWS*_ = 10.6/h). However, hypoxic burden during SWS was significantly reduced with oxygen compared to without oxygen [*OSA_*SWS*_* + *O*_2_ = 23 (%min)/h, *OSA_*SWS*_* = 37 (%min)/h]. No significant OSA was observed in non-rapid eye movement (REM) stage 1 (NREM 1), non-REM stage 2 (NREM 2), or REM sleep (e.g., non-SWS) in any condition. The SWS-specific CPAP withdrawal induces OSA with SF and IH. The addition of oxygen during CPAP withdrawal results in SF with significantly less severe hypoxemia during the induced respiratory events in SWS. This model of SWS-specific CPAP withdrawal disrupts SWS with a physiologically relevant stimulus and facilitates the differentiation of SF and IH in OSA.

## Introduction

In obstructive sleep apnea (OSA), the cardinal polysomnographic (PSG) features following partial or complete closure of the airway are sleep fragmentation (SF) and intermittent hypoxemia (IH). Greater OSA severity generally results in more profound sleep disruption and less time spent in slow-wave sleep (SWS), which is observed to rebound following continuous positive airway pressure (CPAP) treatment (Fietze et al., 1997; Brillante et al., 2012). Sleep disruption can alter the metabolism of proteins prone to aggregation, such as amyloid-β (Aβ) and tau that promote subsequent neurodegeneration observed in Alzheimer’s disease (AD) (Ju et al., 2017; Olsson et al., 2018). There is a theorized bidirectional relationship between sleep disruption and neurodegeneration (Ju et al., 2014; Musiek et al., 2015; Andrade et al., 2018) with the magnitude of sleep changes in aging, specifically electroencephalographic (EEG) slow-wave activity (SWA) by which SWS is defined, being associated with the degree of hippocampal-dependent memory impairments (Westerberg et al., 2012; Mander et al., 2013; Varga A.W. et al., 2016), and the severity of dementia risk assessed by Aβ (Mander et al., 2015; Varga A. et al., 2016). Although these observations implicate SWS in both memory and the regulation of Aβ in the brain, a causal role for SWS in each of the processes would be strengthened by disrupting SWS and measuring outcomes against normally consolidated SWS within subjects. While the stage-specific sleep disruption has been achieved with auditory tones (Tasali et al., 2008; Van Der Werf et al., 2011), comparing the disruption of sleep from OSA while attenuating hypoxemia may represent a more physiological method of testing the within-subject consequences of SWS disruption.

Understanding the mechanisms by which OSA might mediate deleterious health effects would benefit not only from evaluating sleep stage specificity but also from disentangling the individual contributions of SF and IH. Both SF and IH can increase sympathetic activation, but oxidative stress, likely more prominent in IH, is needed for the expression of inflammatory stress markers and endothelial dysfunction (Lavie, 2009; Hoyos et al., 2015). Novel measures capturing the severity of IH such as hypoxic burden, which encapsulates the area under the oxygen saturation by pulse oximetry (SpO_2_) curve, demonstrate a better predictive capacity than the Apnea-Hypopnea Index (AHI) for OSA-associated cardiovascular disease-related mortality (Azarbarzin et al., 2019) and impairments in psychomotor vigilance test (PVT) performance (Kainulainen et al., 2020).

There is increasing evidence of impaired sleep-dependent memory processing with OSA (Djonlagic et al., 2014; Landry et al., 2014; Mullins et al., 2021), with some evidence suggesting that the OSA effect is mediated by changes to SWS duration or power (Djonlagic et al., 2021), factors more likely influenced by SF. A better understanding of the physiological vulnerability of an individual to sleep disruption caused by SF and IH in OSA is foundational to the personalized treatment approaches and disease risk assessment in sufferers (Mullins et al., 2020).

A possible way to simultaneously address OSA stage-specificity and to disentangle the SF and IH associated with OSA is to employ a model of CPAP withdrawal in individuals with established OSA that are adherent to CPAP. While several studies have used CPAP withdrawal for short and long periods to investigate the recurrence of OSA and its effects (Phillips et al., 2007; Kohler et al., 2011; Jun et al., 2016), none to our knowledge have triggered the intervention using sleep stage. Our approach in this study builds on prior studies from our group with rapid eye movement (REM)-specific sleep disruption through CPAP withdrawal (Varga et al., 2014). By limiting CPAP withdrawal to SWS through real-time EEG monitoring, we aimed to recapitulate OSA in SWS only, with negligible OSA in other sleep stages. By withdrawing CPAP selectively during SWS, either with or without oxygen preceding the withdrawal, the contributions of SF with and without IH can be assessed during a sleep state important for the normal processing of memory and AD biomarker fluid metabolites.

We hypothesized that selective CPAP withdrawal during SWS will result in SWS fragmentation and oxygen desaturation, while the addition of supplemental oxygen will mitigate the IH while maintaining SF caused by the induced OSA. Demonstrating this lends further support to the use of sleep stage-specific CPAP withdrawal in OSA as a means to investigate a variety of health-related outcomes.

## Materials and Methods

### Participants

In total, 26 subjects with moderate-to-severe OSA (AHI4% ≥ 20), who were compliant with therapeutic CPAP (≥ 4 h/night for ≥ 70% of nights in the last month), were recruited as part of two studies as follows: first, a pilot study investigating the effect of SWS disruption (using the intervention described in this study) on overnight spatial navigational memory processing, and a subsequent, ongoing study investigating the effects of this SWS intervention on overnight memory and AD fluid biomarkers. All subjects signed informed consent documents, and the protocols were approved by the Mount Sinai Institutional Review Board. The exclusion criteria for both studies included being older than 85 years, the chronic use of any sedative, stimulant, or neuroleptic drug, diagnosis of a sleep disorder other than OSA or the presence of other critical comorbid conditions, preexisting cognitive or neurological deficits, proneness to vertigo, pregnancy, or scheduled surgical weight loss during the study duration. Two participants were excluded from this analysis, i.e., one from the pilot study who had low baseline SpO_2_ (∼ 90%) during the CPAP condition, and one from this study due to the presence of previously undiagnosed chronic obstructive pulmonary disease. The demographics and diagnostic OSA severity measures of the participants are presented in Table 1. Although the eligibility criteria were confirmed at the time of enrollment, diagnostic AHI4% and/or AHI3A metrics were unavailable for three participants because level 3 (without EEG) diagnostic studies were used.

**TABLE 1:** Demographics and sleep macrostructure of the participants.

*N* = 24					
Age	Years	55 (17)			
Sex	Female	29%			
BMI	kg/m^2^	33.8 (7.4)			
Diagnostic AHI4% (*N* = 22)	/h	41.2 (29.0)			
Diagnostic AHI3A/RDI (*N* = 22)	/h	55.8 (21.9)			

		**CPAP**	**OSA_SWS_**	**OSA_SWS_ + O_2_**	***p*-value**

Total sleep time (TST)	h	6.6 (1.0)	6.6 (0.8)	7.1 ± 1.2	n.s.
Sleep efficiency (SE)	% TST/TIB	88.0 (8.8) *^b^*	88.6 ± 8.4	92.6 (6.7)	0.04
Sleep onset latency (SOL)	min	4.4 (5.2)	4.2 (5.8)	3.1 (7.0)	n.s.
Wake after sleep onset (WASO)	min	47.0 (41.9)	31.8 (50.3)	28.8 (36.2)	n.s.
NREM 1	%TST	10.4 (4.3)	9.4 (4.9)	8.7 (5.8)	n.s.
NREM 2	%TST	51.6 ± 7.1	54.1 ± 8.5	54.1 ± 6.4	n.s.
NREM 3	%TST	16.2 (5.9) *^a,b^*	13.8 (10.0)	13.1 (5.6)	0.01
REM	%TST	21.4 ± 5.8	20.4 ± 5.9	21.9 ± 5.2	n.s.

*AHI4%, apnea-hypopnea index (hypopnea 4% O_2_ desaturation criteria); AHI3A, apnea hypopnea index [hypopnea 3% O_2_ desaturation/electroencephalographic (EEG) arousal criteria]; TST, total sleep time; REM, rapid eye movement; NREM 1, non-REM stage 1; NREM 2, non-REM stage 2; SWS, slow-wave sleep.*

*^a^Significant difference between continuous positive airway pressure (CPAP) and OSA_SWS_ conditions.*

*^b^Significant difference between CPAP and OSA_SWS_ + O2 conditions; n.s., not significant. Values expressed as mean ± SD or median (interquartile range). All significance levels are for the Friedman or one-way repeated measures ANOVA tests.*

### Slow-Wave Sleep Continuous Positive Airway Pressure Withdrawal and Supplemental Oxygenation Intervention

Participants attended three, non-consecutive overnight PSG studies at the Mount Sinai Integrative Center for Sleep in New York, NY, United States, and a full-night PSG on therapeutic CPAP was performed following the standard American Academy of Sleep Medicine (AASM) criteria (Iber et al., 2007). There were three PSG conditions presented in a randomized order as follows: (1) consolidated sleep with CPAP held at therapeutic pressure (*CPAP*); (2) CPAP withdrawn exclusively in SWS (*OSA*_*SWS*_), and (3) CPAP withdrawn exclusively in SWS with simultaneous addition of supplemental oxygen (*OSA_*SWS*_* + *O*_2_). On the SWS-disruption nights (*OSA*_*SWS*_ and *OSA_*SWS*_* + *O*_2_), a sleep technician lowered CPAP to 4 cmH_2_O as soon as 2 consecutive slow oscillations of EEG (≤ 1 Hz) were identified or the EEG indicated SWS. CPAP was returned to the therapeutic pressure value on arousal from sleep (i.e., microarousal or wake). During instances in which CPAP withdrawal to 4 cmH20 was insufficient to disrupt SWS, CPAP was further reduced to 0 cmH_2_O, using equipment and techniques we developed previously to deliver CPAP of 0 cmH_2_O without rebreathing (Patel et al., 2011). On the SWS-disruption nights with oxygen (*OSA_*SWS*_* + *O*_2_), the CPAP withdrawal was as described earlier. In addition, a sleep technician delivered between 1 and 4 L/min oxygen, individually titrated starting at 1 L/min, *via* oxygen tubing connected to the CPAP circuit of the participants as soon as 2 consecutive slow oscillations of EEG were identified or the EEG indicated SWS. O_2_ delivery was ended on arousal from sleep (i.e., microarousal or wake). To account for considerable variability in participant response to both pressure withdrawal and oxygen therapy, the intervention parameters (i.e., timing of CPAP withdrawal and oxygen flow) were individually titrated and determined in real-time for each participant during each PSG. All study interventions were conducted by both, or one of two registered polysomnographic technologist (RPSGTs) (BC and ZR). When comparing the number of CPAP withdrawals between the two operators, there were no significant differences.

### Polysomnography

Participants were connected to PSG equipment relative to their habitual bedtime. A bedtime questionnaire was completed that recorded information such as caffeine, alcohol, food, and drug intake for that day. A full-night PSG was acquired following the standard AASM criteria (Iber et al., 2007) at the Mount Sinai Integrative Sleep Center using Compumedics E-series and Grael 2 systems (Melbourne, VIC, Australia). The signal acquisition included EEG (minimum F3, F4, C3, C4, O1, O2, M1, and M2), left and right electrooculography (EOG), all sampled at 256 Hz and referenced to a common patient and ground electrode, submental bipolar EMG, bilateral tibialis anterior bipolar EMG, respiration by a pressure transducer and PAP device interface, effort by rib/abdomen impedance plethysmography, single-channel ECG, and SpO_2_. PSGs were scored in 30-s epochs according to the standard criteria (Berry et al., 2012) for sleep and EEG arousals. Total sleep time and percent time spent in wake, REM sleep, non-REM stage 1 (NREM 1), non-REM stage 2 (NREM 2), and SWS were determined. Respiratory events were scored from the airflow signal using the AASM criteria, and the stage-specific (Total, REM, and NREM) apnea indices were calculated. Apneas were defined as the absence of airflow for ≥ 10 s. Hypopnea 4% was defined as a reduction in the amplitude of breathing by 30% or more for ≥ 10 s with ≥ 4% decline in blood oxygen saturation, irrespective of the presence of arousal. Hypopnea (3% or arousal) was defined as a reduction in the amplitude of breathing by 30% or more for ≥ 10 s accompanied by ≥ 3% decline in blood oxygen saturation or EEG arousal. The AHI4% was defined as the sum of all apneas and hypopneas with ≥ 4% desaturation divided by the total sleep time in hours. The AHI3A was defined as the sum of all apneas and hypopneas with ≥ 3% desaturation or EEG arousal divided by the total sleep time in hours. All studies were scored by one of two RPSGTs (BC and ZR).

Sleep fragmentation during SWS was assessed in three ways as follows: (1) the arousal index was calculated by dividing the total number of arousals by the duration of sleep (arousals/h), (2) average sleep bout length was calculated from the hypnogram by assessing the mean length in minutes of contiguous epochs (30 s) of sleep scored as SWS, terminated by one or more epochs scored as another stage, such as wake, and (3) the survival curve analysis for sleep continuity was performed for SWS using a bootstrap-based technique that accounts for the number of contiguous epochs (30 s) contributed by each subject in each condition (Kishi et al., 2011; Varga et al., 2014).

Hypoxemia during SWS was assessed in five ways: (1) lowest SpO_2_ desaturation in the 30 s following an apnea/hypopnea; (2) the Oxygen Desaturation Index (ODI), which is defined as the number of SpO_2_ desaturations ≥ 3% divided by the duration of sleep; (3) absolute SpO_2_ nadir, (4) time spent with SpO_2_ below 90% and (5) hypoxic burden, defined as percentage minute per hour of SWS. The hypoxic burden was calculated using MATLAB R2020a (MathWorks, Natick, MA, United States). In brief, for every event with desaturation ≥ 3%, the area between the baseline and the SpO_2_ trace was calculated. The sum of all such areas was defined as the hypoxic burden. The baseline for each event was determined as the inflection point preceding a SpO_2_ nadir. Values were normalized using a square root transform.

The OSA severity measures, arousal index, and SpO_2_ nadir are also reported for non-SWS and during total sleep time.

### Data Analysis

Data were analyzed and plotted using MATLAB R2020a (MathWorks, Natick, MA, United States) and R Studio version 1.1.456 using the package ggplot2 (Wickham, 2016). The three PSG conditions, namely, *CPAP*, *OSA*_*SWS*_, and *OSA_*SWS*_* + *O*_2_ were compared on the measures of OSA severity (AHI4% and AHI3A), SF (arousal index, average bout length, and sleep continuity), and IH (lowest event-related SpO_2_, ODI 3%, SpO_2_ time below 90%, and hypoxic burden) during SWS. OSA severity measures, arousal index, and SpO_2_ Shapiro–Wilk test statistics were used to assess the normality of the data. For the non-normally distributed data, median and interquartile range values are reported, and for the normally distributed data, mean and SD values are reported. As appropriate, the Friedman or one-way repeated measures ANOVA tests were performed to compare the PSG conditions. When the significant difference between the PSG conditions was found, Nemenyi or Tukey’s *post hoc* tests were used to test for differences between PSG condition pairs. The Kolmogorov–Smirnov tests were used to compare the survival curve distributions of cumulative SWS bout lengths between the three conditions. Differences in IH measures between *OSA*_*SWS*_ and *OSA_*SWS*_* + *O*_2_ conditions were investigated using paired Wilcoxon or *t*-tests, as appropriate. The results for all the statistical analyses were considered significant at *p* < 0.05.

## Results

### Demographics and Sleep Macrostructure

The mean age of the 24 participants was 55 ± 17 years, and 7 (29%) were female. Participants were overweight on average with a mean body mass index (BMI) = 33.8 ± 7.4 kg/m^2^. Participants had moderate-to-severe OSA according to either the AHI4% or AHI3A definition at diagnosis.

The PSG macrostructure variables were within the age-expected range in all three PSG conditions. %SWS was significantly reduced during both CPAP withdrawal conditions [*OSA*_*SWS*_ = 13.8 (10.0)%, *OSA_*SWS*_* + *O*_2_ = 13.1 (5.6)] compared to *CPAP* [16.2 (5.9)%]. Sleep efficiency was significantly greater during CPAP withdrawal with oxygen [*OSA_*SWS*_* + *O*_2_ = 92.6 (6.7)%] compared to *CPAP* = 88.0 (8.8)%, as shown in Table 1.

### Execution of Polysomnographic Interventional Conditions

There was no significant difference between the number of CPAP withdrawals during the *OSA_*SWS*_ and OSA_*SWS*_ + O_2_* conditions [*OSA_*SWS*_ = 22 (15), OSA_*SWS*_* + *O_2_* = 27 (23)]. The order of the presentation of the 3 interventional conditions for these 24 participants was randomized. The distribution of the first-night conditions was as follows: *OSA*_*SWS*_ = 11, *OSA_*SWS*_* + *O*_2_ = 2, *CPAP* = 11. The median time between PSG conditions/visits was 25.5 days (range: 1–30 weeks).

### Effects of Intermittent Continuous Positive Airway Pressure Withdrawal During Slow-Wave Sleep With and Without Supplemental Oxygen on Sleep Fragmentation and Intermittent Hypoxemia

The effect of the PSG intervention on the measures of OSA severity, SF and IH during SWS, and during all sleep are displayed in Table 2. The number of times CPAP was withdrawn during SWS did not differ significantly between with and without oxygen conditions [*OSA_*SWS*_* + *O*_2_ = 27 (23), *OSA*_*SWS*_ = 22 (15)].

**TABLE 2:** Obstructive sleep apnea (OSA) severity, sleep fragmentation, and intermittent hypoxemia.

During SWS		CPAP	OSA_*SWS*_	OSA_*SWS*_ + O_2_	*p*-value
# of CPAP withdrawals	SWS	0 (0)^a^^,^^b^	22 (15)	27 (23)	<0.0001
AHI3A	SWS	0.0 (0.9)^a^^,^^b^	18.0 (11.3)	13.5 (11.5)	<0.0001
AHI4%	SWS	0.0 (0.0)^a^^,^^b^	9.6 (13.0)^c^	3.5 (5.7)	<0.0001
Arousal index	p/h SWS	1.1 (2.3)^a^^,^^b^	10.6 (8.6)	10.7 ± 6.3	<0.0001
Average sleep bout	min SWS	5.9 (4.9)^a^^,^^b^	2.1 (1.2)	2.4 (1.4)	<0.001
Lowest event-related SpO_2_	% in SWS	–	86.3 ± 5.8^c^	90.5 ± 3.7	<0.001
Event duration	s	–	24.3 (7.1)	26.8 (10.0)	n.s.
ODI 3%	SWS	–	22.8 ± 11.3^c^	14.2 ± 10.8	<0.0001
SpO_2_ time below 90%	% of SWS	–	0.8 (1.3)^c^	0.2 (0.4)	<0.01
Hypoxic burden	(% min) h SWS	–	37.5 (19.3)^c^	22.6 ± 10.1	<0.001
SpO_2_ nadir	% in SWS	–	88.6 ± 6.3^c^	90.6 ± 3.7	<0.001
**Non-SWS (NREM1 + NREM2 + REM)**					
AHI3A	Non-SWS	1.2 (3.8)	2.1 (6.5)	2.4 (5.1)	n.s.
AHI4%	Non-SWS	0.8 (2.2)^a^^,^^b^	2.1 (5.9)	2.3 (2.8)	<0.001
Arousal index	p/h non-SWS	11.8 ± 6.0	10.3 (8.2)	9.6 (4.6)	n.s.
SpO_2_ nadir	% non-SWS	88.9 ± 2.9	87.3 (8.3)	89.4 (4.3)	n.s.
**All sleep**					
AHI3A	TST	1.0 (3.3)^a^^,^^b^	5.2 (4.2)	4.0 (4.8)	<0.0001
AHI4%	TST	0.7 (1.9)^a^^,^^b^	3.4 (3.6)	2.7 (2.7)	<0.0001
Arousal index	p/h TST	10.7 ± 5.0	10.7 (7.6)	9.7 ± 4.1	n.s.
SpO_2_ nadir	% in TST	88.4 ± 3.9^a^	86.0 (6.3)^c^	89.0 (5.3)	<0.001

*AHI4%, apnea-hypopnea index (hypopnea 4% O_2_ desaturation criteria); AHI3A, apnea hypopnea index (hypopnea 3% O_2_ desaturation/EEG arousal criteria); SpO_2_, peripheral capillary oxygen saturation; TST, total sleep time; TSP, total sleep period; REM, rapid eye movement; NREM 1, non-REM stage 1; NREM 2, non-REM stage 2; SWS, slow-wave sleep.*

*^a^Significant difference between CPAP and OSA_SWS_ conditions.*

*^b^Significant difference between CPAP and OSA_SWS_ + O2 conditions.*

*^c^Significant difference between OSA_SWS_ and OSA_SWS_ + O2 conditions; n.s., not significant. Values expressed as mean ± SD or median (interquartile range). All p-values denote the Friedman or one-way repeated measures ANOVA tests when three conditions were compared and represent paired t-tests or Wilcoxon tests when two conditions are compared.*

#### Sleep Fragmentation

**FIGURE 1:**
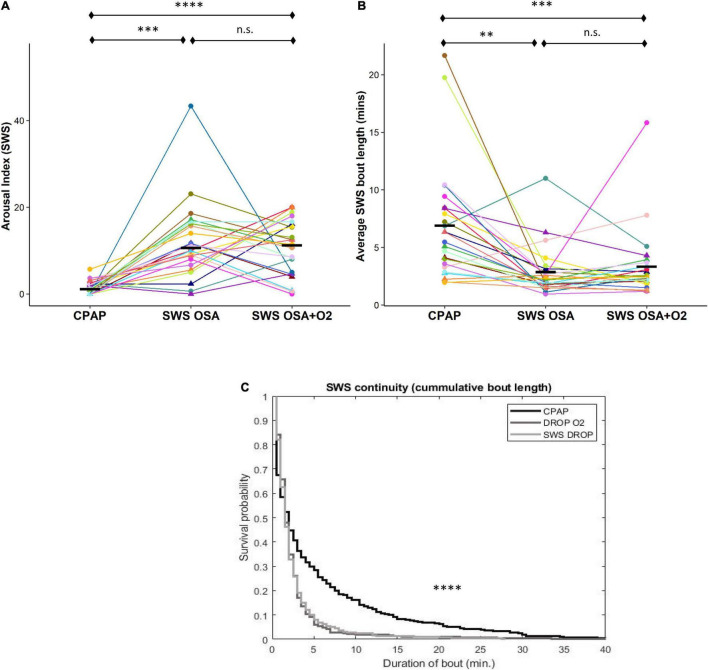
Intermittent withdrawal of continuous positive airway pressure (CPAP) during slow-wave sleep (SWS) results in increased arousals, shorter sleep bouts, and less sleep continuity. Sleep fragmentation (SF) during SWS, according to the arousal index (a), was significantly greater during *OSA*_*SWS*_ and *OSA_*SWS*_* + *O2* conditions compared to CPAP. Significantly reduced sleep continuity, corresponding to shorter mean (b) and cumulative bout lengths (c), was also observed during *OSA*_*SWS*_ (light gray) and *OSA_*SWS*_* + *O2* (dark gray) compared to the CPAP (black line) condition. In (a,b), all 24 participants are represented by different colors with a triangle indicating female. Median values for each condition are shown as a solid black line. There were no statistically significant differences between *OSA*_*SWS*_ and *OSA_*SWS*_* + *O2* for any measure of SF. n.s., not significant; ^****^p < 0.0001, ^***^p < 0.001, ^**^p < 0.01 and *p < 0.05.

The arousal index during SWS was significantly greater during both CPAP withdrawal conditions compared to CPAP [*OSA_*SWS*_* = 10.6 (8.6), *OSA_*SWS*_* + *O*_2_ = 10.7 ± 6.3, *CPAP* = 1.1 (2.3), and *p* < 0.00001] (Figure 1A). The average sleep bout length (i.e., measure of sleep continuity) was reduced during both CPAP withdrawal conditions compared to CPAP [*OSA_*SWS*_* = 2.1 (1.2) min, *OSA_*SWS*_* + *O*_2_ = 2.4 (1.4) min, *CPAP* = 5.9 (4.9) min, and *p* < 0.0001 (Figure 1B). The cumulative duration probability distribution for SWS in the *CPAP* condition was significantly right-shifted (*p* < 0.0001), indicating larger cumulative bout durations and less SWS fragmentation in participants during the *CPAP* condition compared to the CPAP withdrawal conditions (Figure 1C). Importantly, no significant differences were observed between the CPAP withdrawal conditions (with or without oxygen) for any measures of SF tested.

#### Intermittent Hypoxemia

**FIGURE 2:**
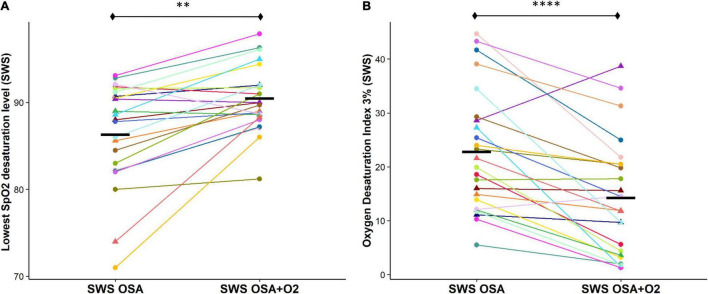
Supplemental O_2_ during CPAP withdrawal results in less OSA event-related SpO_2_ desaturation. The OSA event-related SpO_2_ desaturation is significantly reduced with the addition of supplemental oxygen as shown by an elevated lowest desaturation level during SWS **(A)** and a reduced number of oxygen desaturations ≥ 3% per hour of SWS **(B)**. All 24 participants are represented by different colors with a triangle indicating female. Mean values for each condition are indicated with a solid black line. n.s., not significant; ^****^p < 0.0001, ^***^p < 0.001, ^**^p < 0.01 and *p < 0.05.

Lowest OSA event-related SpO_2_ desaturation during SWS was significantly reduced during the CPAP withdrawal with oxygen condition compared to without oxygen condition [*OSA_*SWS*_* + *O_2_* = 90.5 ± 3.7%, *OSA*_*SWS*_ = 86.3 ± 5.8%, *p* < 0.01] (Figure 2A). The ODI (3%) was also significantly reduced during the CPAP withdrawal with oxygen condition compared to without oxygen condition [*OSA_*SWS*_* + *O*_2_ = 14.2 ± 10.8, *OSA*_*SWS*_ = 22.8 ± 11.3, and *p* < 0.01] (Figure 2B).

**FIGURE 3:**
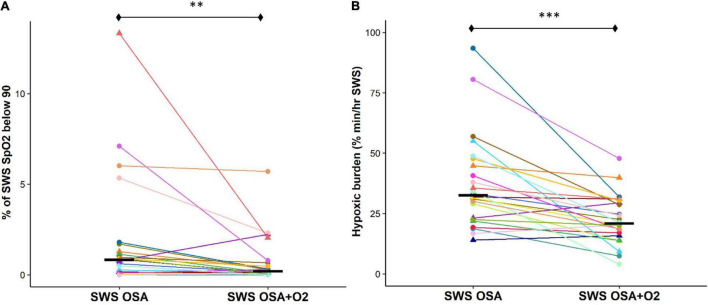
Supplemental O_2_ during CPAP therapy withdrawal results in a less cumulative SpO_2_ desaturation during SWS. Cumulative SpO_2_ desaturation is significantly reduced with the addition of supplemental oxygen as shown by a reduced time during SWS when SpO_2_ values were below 90% **(A)** and hypoxic burden measures are lower **(B)**. All 24 participants are represented by different colors with a triangle indicating female. Median values for each condition are shown as a solid black line. n.s., not significant; ****p < 0.0001, ***p < 0.001, **p < 0.01 and *p < 0.05.

Percentage time SpO_2_ that was below 90% during SWS was also significantly reduced during the CPAP withdrawal with oxygen condition compared to without oxygen condition [*OSA_*SWS*_* + *O_2_* = 0.2 (0.4), *OSA_*SWS*_* = 0.8 (1.3), and *p* < 0.01] (Figure 3A). Importantly, the hypoxic burden during SWS was significantly reduced during the CPAP withdrawal with oxygen condition compared to without oxygen condition [*OSA_*SWS*_* + *O_2_* = 22.6 ± 10.1 (%min)/h, *OSA_*SWS*_* = 37.5 (19.3) (%min)/h, and *p* < 0.001] (Figure 3B).

#### Obstructive Sleep Apnea Severity

**FIGURE 4:**
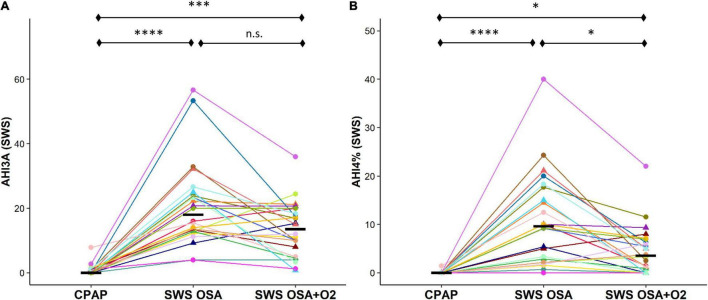
Intermittent withdrawal of CPAP therapy during SWS results in an increase in OSA severity measures. OSA severity during SWS, as measured by AHI3A **(A)** and AHI4% **(B)**, was significantly greater during *OSA*_*SWS*_ and *OSA_*SWS*_* + *O2* conditions compared to CPAP. All 24 participants are represented by different colors with a triangle indicating female. Median values for each condition are shown as a solid black line. i.e. n.s., not significant; ****p < 0.0001, ***p < 0.001, **p < 0.01 and *p < 0.05.

The OSA severity during SWS (AHI3A definition) was significantly higher during both the CPAP withdrawal conditions compared to CPAP [*OSA_*SWS*_* = 18.0 (11.3)/h, *OSA_*SWS*_* + *O_2_* = 13.5 (11.5)/h, *CPAP* = 0.0 (0.9)/h, *p* < 0.0001]. There were no significant differences in AHI3A measures between the *OSA*_*SWS*_ and *OSA_*SWS*_* + *O*_2_ conditions (Figure 4A). The OSA severity during SWS (AHI4 definition) was significantly higher during both the CPAP withdrawal conditions compared to CPAP [*OSA*_*SWS*_ = 9.6 (13.0)/h, *OSA_*SWS*_* + *O*_2_ = 3.5 (5.7)/h, *CPAP* = 0.0 (0.0)/h, *p* < 0.0001]. However, AHI4% was significantly lower with supplemental O_2_ than without (Figure 4B).

The CPAP withdrawal in SWS resulted in no significant change in the AHI3A in non-SWS stages and small but statistically significant increases in the AHI4% during non-SWS sleep stages, although all values remained in the normal range, suggesting the absence of clinical disease (AHI4% < 5/h; AHI3A < 15/h). There were no significant differences observed between the duration of respiratory events during the *OSA*_*SWS*_ compared to *OSA_*SWS*_* + *O*_2_ conditions.

## Discussion

Using a within-subject model of SWS-specific CPAP withdrawal and supplemental oxygen across three different PSG conditions, we were able to successfully recapitulate OSA during SWS, achieving SWS fragmentation (i.e., increased EEG arousals and reduced sleep continuity) in both CPAP withdrawal conditions while significantly reducing IH (i.e., less SpO_2_ desaturations and lower hypoxic burden) in the condition with supplemental oxygen. Furthermore, SF was equivalent in both CPAP withdrawal conditions, and IH was significantly reduced with the addition of supplemental oxygen prior to the CPAP drop, according to several PSG measures of peripheral oxygenation and the AHI4%. The induction of OSA was specific to SWS based on real-time EEG monitoring. Although there were small increases in the measures of OSA severity (AHI4%) in non-SWS sleep stages compared to the control CPAP night, these were significantly smaller than the increases in OSA severity observed in SWS, and the absolute severity of OSA in the non-SWS stages was in a range considered clinically normal. These results confirm that, on average, participants are responding to the experimental intervention as intended and demonstrate a novel method of achieving SWS fragmentation without significant IH. This permits increased certainty when it comes to the interpretation of whether limiting hypoxemia during OSA-disrupted SWS affects any of the variety of health outcomes considered to depend on SWS.

We observed significantly less SWS as a percentage of total sleep time during both CPAP withdrawal conditions compared to CPAP. We additionally assessed the effect of CPAP withdrawal during SWS by reporting SWS bout length in minutes. This metric describes the average duration of contiguous SWS scored before either awakening or a shift to another stage of sleep. We observed significantly shorter SWS bout lengths during both CPAP withdrawal conditions compared to CPAP, suggesting increased SWS fragmentation in addition to the reduced overall amount. Beyond %SWS, there were no significant differences in other sleep stages (e.g., NREM or REM) between conditions. We did observe significantly higher sleep efficiency during *OSA_*SWS*_* + *O*_2_, possibly due to the distribution of first-night PSGs being lower for that condition.

In the current paradigm, we elected to induce OSA to fragment SWS due to the importance of SWS in outcomes such as memory (Varga A.W. et al., 2016; Djonlagic et al., 2021), metabolism of AD biomarkers (Mander et al., 2013; Varga A. et al., 2016; Ju et al., 2017), hormone secretion (Sassin et al., 1969; Van Cauter et al., 1997), and glucose metabolism (Tasali et al., 2008; Ukraintseva et al., 2020), but the approach can be modified to target other sleep stages and may even be able to be automated by using neural net-based sleep stage classifiers (Kam et al., 2020) in a closed-loop system. One limitation of inducing OSA in SWS is that a majority of patients show a considerable reduction in OSA severity during SWS compared to lighter NREM and REM sleep (Ratnavadivel et al., 2009), at least in part because apneas/hypopneas are associated with fewer visually detected cortical arousals during SWS (Dingli et al., 2002). Despite this, we were still capable of achieving SWS-specific SF and IH using our model of CPAP withdrawal.

Although SWS is observed to attenuate with age (Carrier et al., 2001; Ohayon et al., 2004), and less so in women (Carrier et al., 2011), it is not known how this potentially interacts with the expression of OSA during SWS. There are well-characterized sex differences in OSA physiological traits in older individuals, with women displaying lower AHI levels, less airway collapsibility, a lower threshold to arousal from apnea-hypopnea events, and a more stable ventilatory control system during NREM sleep than men (Won et al., 2020). It is not known if these observed sex differences in OSA traits during NREM are different during NREM2 compared to SWS specifically. It would be possible to investigate this with this model, but due to the small number of women in our sample, we were not powered to explicitly assess this.

Another factor that can influence the capacity to effectively recapitulate OSA *via* CPAP withdrawal (both within and between subjects) is variability in sleeping body position. Notably, previous research indicates at least a two-fold worsening of the AHI in approximately two-thirds of patients in the supine position (Cartwright, 1984; Eiseman et al., 2012), and one study showed a five-fold worsening when both trunk and head were in the supine position (van Kesteren et al., 2011). Regardless, and despite not controlling for body position in this analysis, we were able to demonstrate that, on average, pretreatment with supplemental oxygen significantly limited several measures of hypoxemia in response to OSA.

Other factors that may influence the response of an individual to the intervention include changes in weight and/or therapy effectiveness between PSG visits. Significant weight loss with surgery is known to change the requirements of the pressure therapy (Lankford et al., 2005). Although planned bariatric surgery was exclusionary for participants in this analysis, it is plausible that other causes of weight loss would reduce the expected severity of OSA from CPAP withdrawal in comparison with the initial diagnostic sleep study of an individual or between research nights, which was 25.5 days on average and ranged from 1 to 30 weeks. Conversely, pressure inadequacy could have arisen between visits for reasons such as increases in weight or reduced mask fit (Kakkar and Berry, 2007), but all subjects included in the analysis were confirmed to be fully treated on the control night and treated in all stages outside of SWS on the intervention nights.

Oxygen therapy has been used as an alternative to CPAP to treat OSA, and despite reducing hypoxemia, it is inferior to CPAP in both reducing AHI and improving most clinical outcomes (Zeineddine et al., 2021). The degree to which non-titrated oxygen can prevent apnea/hypopnea-associated desaturations likely depends on factors such as the duration of pretreatment, the degree of airway closure during an event, and the duration of the event. Oxygen therapy may also increase the length of apnea-hypopnea events (Mehta et al., 2013). We did not observe a shortening of event duration on O_2_, likely due to the short duration and intermittent nature of our oxygen flow delivery. Debate remains about the effect of O_2_ on blood pressure with some research indicating that O_2_ therapy improves hypertension (Turnbull et al., 2019) and others indicating that it does not (Gottlieb et al., 2014). Studies examining the effect of supplemental oxygen in OSA have delivered the oxygen variably using nasal cannulas and through masks in sham PAP conditions, but no study has actively manipulated the oxygen flow and PAP simultaneously within an individual sleep stage as demonstrated in this study. When the supplemental oxygen is delivered across the full duration of sleep, the measures of hypoxic burden are generally reduced while the measures of SF are generally unchanged (Loredo et al., 2006; Turnbull et al., 2019). Our data are consistent with these observations.

Slow wave sleep is a stage of sleep with highly stable breathing (Ratnavadivel et al., 2009), implying low loop gain. Previous literature has suggested that low loop gain predicts poorer responsiveness to oxygen therapy in terms of AHI reduction (Wellman et al., 2008; Edwards et al., 2014; Sands et al., 2018). Therefore, one would predict less change in the AHI in response to supplemental oxygen when CPAP is withdrawn during SWS. Our findings that were showing no significant differences in the AHI3A in SWS with and without O_2_ are consistent with these previous observations.

Carbon dioxide (CO_2_) levels are a driver of ventilation and arousal not routinely monitored in adult PSG. In this study, we did not measure CO_2_, but it is plausible that the response of some individuals to the CPAP withdrawal with supplemental oxygen was influenced in part by changing the CO_2_ levels. However, the effect of such short duration, low flow O_2_ delivery on CO_2_ levels is not known, and we did not observe significant differences in arousal index between the *OSA*_*SWS*_ and *OSA_*SWS*_* + *O*_2_ conditions.

## Conclusion

We have implemented a tool for the stage-specific disruption of sleep with OSA with the simultaneous ability to significantly limit hypoxemia and hypoxemic burden. We feel such a tool will be useful toward understanding the causal contributions of individual sleep stages toward a variety of health outcomes while disentangling the contributions of SF and IH from OSA.

## Data Availability Statement

The raw data supporting the conclusions of this article will be made available by the authors, without undue reservation.

## Ethics Statement

The studies involving human participants were reviewed and approved by Icahn School of Medicine at Mount Sinai Program for the Protection of Human Subjects. The patients/participants provided their written informed consent to participate in this study.

## Author Contributions

AEM: data curation, tabulation, analysis, and manuscript writing. AP: data analysis and manuscript editing and review. KK: data analysis and manuscript review. BC: data collection and analysis. ZR: data collection, analysis, and manuscript review. AF, DV, and RS: data collection and tabulation. TT, JB, AMM, and OB: data collection. DR and IA: theory conceptualization and manuscript editing and review. AV: theory conceptualization, data collection, and manuscript writing, editing, and review. All authors contributed to the article and approved the submitted version.

## Conflict of Interest

The authors declare that the research was conducted in the absence of any commercial or financial relationships that could be construed as a potential conflict of interest.

## Publisher’s Note

All claims expressed in this article are solely those of the authors and do not necessarily represent those of their affiliated organizations, or those of the publisher, the editors and the reviewers. Any product that may be evaluated in this article, or claim that may be made by its manufacturer, is not guaranteed or endorsed by the publisher.
